# Polymer-Wrapped Oil Droplets and Polymer Particles with Complex Curvilinear Polyhedral Geometries by Surfactant-Driven Elastocapillary Buckling of Polymer Capsules

**DOI:** 10.1021/acs.chemmater.5c01556

**Published:** 2025-10-29

**Authors:** Xuanrong Guo, Saverio E. Spagnolie, Nicholas L. Abbott, David M. Lynn

**Affiliations:** † Department of Chemical and Biological Engineering, 5228 University of Wisconsin—Madison, 1415 Engineering Drive, Madison, Wisconsin 53706, United States; ‡ Department of Chemistry, University of Wisconsin—Madison, 1101 University Avenue, Madison, Wisconsin 53706, United States; § Department of Mathematics, University of Wisconsin—Madison, 505 Van Vleck, Madison, Wisconsin 53706, United States

## Abstract

Surfactants have been widely used to tune interfacial tensions in colloidal dispersions, resulting in phenomena such as wetting/dewetting, emulsification, and foaming. In this study, we report that adding micromolar concentrations of surfactants to aqueous dispersions of thin, deformable polymer microcapsules partially filled with droplets of oil induces complex shape changes in both the capsules and the encapsulated oil droplets. Using video microscopy and confocal microscopy, we observed the evolution of complex, anisotropic shapes with apparent six-, five-, four- and 3-fold symmetries in the presence of added surfactant and identified several factors influencing these shape changes and the resulting distribution of shapes, including surfactant concentration and charge, polymer capsule diameter and wall thickness, and the size of the encapsulated oil droplets. Our results suggest that these shape changes are driven by elastocapillarity, in which the addition of surfactant changes the balance between elastic energy and interfacial energy. Furthermore, we show that photopolymerization of deformed droplets of vinyl monomer oils “caged” in these capsules can be used to template the synthesis of polymer particles with complex anisotropic shapes similar to those of the folded and polymer-wrapped oil-filled capsules prior to polymerization. Finally, we demonstrate that the use of degradable polymer cages enables the isolation of “cage-free” polymer particles with complex shapes and geometries.

## Graphical abstract

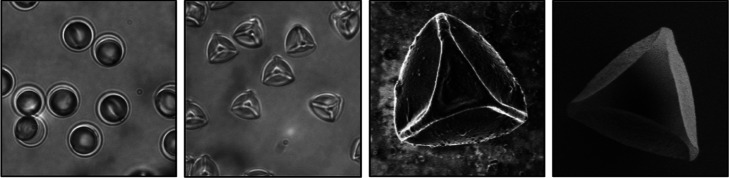

## Introduction

Materials that are soft, reconfigurable, and able to undergo controlled changes in shape are useful in a variety of applications,^1–4^ ranging from wearable electronics^5,6^ and soft robotics^7,8^ to the design of next-generation medical devices^9,10^ and colloidal assemblies with useful optical and/or electromagnetic properties.^11,12^ Past studies have reported a wide range of materials that exhibit simple and complex shape-changing behaviors across a range of length scales and in response to different types of physical, chemical, and/or biological stimuli.^1–12^ At small scales, in particular, interfacial tensions can often dominate over bulk forces, leading to phenomena such as the bending and buckling of elastic sheets as interfacial and elastic energies are minimized.^13–17^ These interactions between capillarity and elasticity, known more generally as elastocapillary interactions,^13–17^ can provide simple, but highly versatile, means to initiate and guide changes in the shapes of synthetic materials, enabling, for example, origami-inspired designs of self-folding three-dimensional structures for microfabrication^13,18,19^ and the encapsulation of liquid droplets through oil-in-water and water-in-oil wrapping.^20,21^ Changes in the balance of interfacial and elastic energies have also been exploited in nature, for example as a mechanism through which basidiomycete fungi can eject spores.^22^ Membrane remodeling by capillary forces has also been observed in liquid–liquid phase separation systems, in which phase-separated droplets appear to play functional roles as though transient intracellular organelles.^23–25^ Such membrane deformations also arise on much larger length scales; for example, insect wings can be deformed by droplets in fog,^26^ and high wavenumber wrinkling and deep folds have even been observed in balloons partially filled with water.^27^

In this paper, we report on unexpected and complex changes in the shapes of microscale oil droplets encapsulated in thin, deformable, and porous polymer shells that occur in the presence of added surfactant. These changes in shape are driven predominantly by changes in the balance of interfacial and elastic energies triggered by the adsorption of surfactant and have potential utility for the design of oil droplets and solid polymer particles with complex shapes and symmetries.

We have reported in past studies on the design of so-called “caged” oil droplets, or thin and semipermeable polymer microcapsule shells partially filled with small, microscale volumes of a hydrophobic oil.^28–31^ Those past studies were focused, in large measure, on the design of caged droplets of thermotropic liquid crystals (LCs) using polymer capsules fabricated by layer-by-layer assembly of the amine-reactive polymer poly­(2-vinyl-4,4-dimethylazlactone) (PVDMA) and the amine-containing polymer poly­(ethylenimine) (PEI).^28–30^ That approach leads to swollen spherical polymer capsules partially filled with smaller spherical (or nearly spherical) caged LC droplets. These caged droplets can undergo changes in shape, capsule wall wetting behavior, and optical properties in the presence of added surfactants that are not observed in bare LC droplets and have potential utility in the context of chemical and biological sensing.^28–30^ During the course of further investigations into the behaviors of these caged LC droplets, we observed droplets caged in certain types of capsules (e.g., larger capsules vs smaller capsules) to undergo large and unexpected transformations in shape when exposed to an anionic surfactant to yield caged oil droplets with complex, anisotropic shapes and apparent six-, five-, four- and 3-fold symmetries.^32^ Additional studies revealed these transformations to be driven by changes in the wetting of the droplets with the walls of the capsules, which lead to large changes in the shapes of the surrounding capsules.

Here, we report on factors that influence these stimuli-responsive shape changes and the resulting distributions of colloidal droplet shapes and test the proposition that these changes in shape reflect a competition between elastic and interfacial effects by exploring changes in polymer capsule size and wall thickness. Our results reveal these transformations to be reversible, with deformed caged oil droplets returning to spherical shapes upon exposure to a cationic surfactant. These transformations also appear to be general; they are observed in “cages” partially filled with a variety of different types of isotropic and anisotropic oils and are driven by changes in the balance between elastic energy and interfacial energy upon contact with surfactant. We further demonstrate that photopolymerization of deformed droplets of vinyl monomers (polymerizable oils) caged in these capsules can be used to template the synthesis of micrometer-scale solid polymer particles with complex anisotropic shapes similar to those of the enclosed oil droplets. Finally, we show that the use of degradable polymer “cages” enables the subsequent removal of the surrounding folded polymer cages and the isolation of “cage-free” anisotropic solid polymer particles with complex curvilinear polyhedral geometries.

## Materials and Methods

### Materials

Branched poly­(ethylenimine) (PEI, *M*_W_ ∼ 25,000), 2,2′-azoisobutyronitrile (AIBN), sodium dodecyl sulfate (SDS), decyltrimethylammonium bromide (DTAB), tetradecane (C14), cystamine dihydrochloride, 1,6-hexanediol dimethacrylate (HDODA), 2,2-dimethoxy-2-phenylacetophenone (DMPAP), Nile Red, tetrahydrofuran (THF), acetonitrile, and acetone were purchased from Sigma-Aldrich (Milwaukee, WI). Benzyl methacrylate (BzMA) was obtained from Scientific Polymer Products (Ontario, NY). 1,4-Dithiothreitol (DTT) was purchased from DOT Scientific (Burton, MI). The thermotropic liquid crystals E7 and 5CB were purchased from Jiangsu Hecheng Display Technology Co., Ltd. (HCCH), China. Ethanol was purchased from Decon Laboratories. 3-(Dimethylamino)­propylamine (99%) was purchased from Acros Organics (New Jersey, USA). FluoroDish confocal dishes were obtained from World Precision Instruments (Sarasota, FL). Glass coverslips were purchased from Fisher Scientific (Pittsburgh, PA). SiO_2_ microparticles (average diameter = 7.96 μm) were purchased from Microparticles GmbH (Berlin, Germany). SiO_2_ microparticles (average diameter = 4.86 μm) were purchased from Bangs Laboratories (Fishers, IN). 2-Vinyl-4,4-dimethylazlactone (VDMA) was a kind gift from Dr. Steven M. Heilmann (3M Corporation, Minneapolis, MN). 1-Aminofluorescein was purchased from Tokyo Chemical Industry (Tokyo, Japan). Poly­(2-vinyl-4,4-dimethylazlactone) (PVDMA, *M*_W_ ∼ 66,626; *D̵* = 4.8) was synthesized by the free-radical polymerization of VDMA, as described previously.^33^ PVDMA labeled with aminofluorescein (5 mol %; referred to from hereon PVDMA_FL_) was synthesized as described previously.^33^ Cystamine free base (Cys) was prepared by deprotection of cystamine dihydrochloride using a previously reported protocol.^33^ Deionization of a distilled water source was performed using a Milli-Q system (Millipore, Bedford, MA) yielding water with a resistivity of 18.2 MΩ. All materials were used as received without further purification unless otherwise noted.

### General Considerations

SiO_2_ microparticles used as substrates for layer-by-layer assembly were rinsed with acetone prior to use. Monomers (BzMA and HDODA) were photopolymerized using an XL-1500 UV Cross-linker (Spectronics Corporation). Bright-field micrographs used for characterization of polymerized particles were acquired using an Olympus IX70 microscope and analyzed using the Metavue version 4.6 software package (Universal Imaging Corporation). All other bright-field micrographs were acquired using an Olympus IX71 inverted microscope (Center Valley, PA) using a 100× oil-immersion objective lens. Bright-field and polarized light micrographs of LC-filled capsules were acquired using a Hamamatsu 1394 ORCAER CCD camera (Bridgewater, NJ) connected to a computer and controlled through Simple PCI imaging software (Compix, Inc., Cranberry Twp., NJ). Laser-scanning confocal microscopy (LSCM) images were acquired using a Nikon A1-R high-speed confocal microscope and processed using Nikon Instruments Software. Scanning electron micrographs were acquired using a LEO 1550VP Field Emission scanning electron microscope (SEM) (Königsallee, Göttingen, Germany). Samples were coated with a thin layer of gold using a sputterer (60 s at 10 mA, 500 V, 70 mTorr) prior to imaging. Zeta potentials were measured using a Malvern Zetasizer Nano ZS.

### Fabrication of Microcapsules

Degradable and nondegradable multilayer microcapsules were fabricated in a layer-by-layer manner similar to previously described methods.^28,33^ The nondegradable polymer PEI was used as an amine-containing building block for the preparation of nondegradable multilayer microcapsules. Briefly, solutions of PEI and PVDMA (or PVDMA_FL_) were prepared in acetone (20 mM with respect to the molecular weight of the polymer repeat unit). SiO_2_ microparticles were placed into plastic microcentrifuge tubes and then rinsed with 1 mL of acetone prior to multilayer assembly. The first layer of PEI was deposited onto the SiO_2_ particles by adding 1 mL of PEI solution to the particle suspension and manually shaking the particles for 1 min to allow sufficient time for the polymer to adsorb on the particle surface. The particles were then centrifuged for 30 s at 6000 rpm. The supernatant was then carefully removed by pipet and the particles were rinsed two times by resuspending them in 1 mL of acetone and vortexing. After each rinse in acetone, the particles were centrifuged for 30 s at 6000 rpm and then the supernatant was removed. The second layer was then deposited on the microparticles by suspending them in 1 mL of PVDMA solution with manual shaking for 30 s to allow sufficient time for the PVDMA to react with primary amines of the deposited PEI layer on the SiO_2_ microparticles. The particles were then rinsed twice with 1 mL of acetone as described above. Subsequent layers were fabricated by repeating this process (by alternately depositing PEI or PVDMA solutions and allowing each layer to react for 30 s) until the desired numbers of PEI/PVDMA layer pairs (or “bilayers”, typically four and a half) were deposited onto the particle surface. After film fabrication, the coated particles were washed with THF and placed into 1 mL of 3-(dimethylamino)­propylamine (DMAPA) in THF (20 mM) and kept on an automated shaker plate for 1 h to react exhaustively with all remaining azlactone functional groups and install tertiary amine functionality. Coated particles were then rinsed with THF three times, dispersed in 1 mL of deionized water, and then treated with hydrofluoric acid (HF, 5.0 M) for 10 min to etch away the silica cores [WARNING: HF solutions and vapors are extremely poisonous and corrosive, and may cause extreme burns that are not immediately painful! Handle with extreme caution, in a chemical fume hood, and using appropriate protective equipment (gloves, face/eye protection, lab coat, etc.), and neutralize waste appropriately. Do not store in glass containers.]. The resulting empty polymer capsules were washed, centrifuged, and resuspended in water five times prior to characterization. To prepare redox-degradable microcapsules, the disulfide-containing diamine cross-linker Cys was used as an amine-containing building block instead of PEI. Solutions of Cys (10 mM, also containing an equimolar amount of DMAPA) and PVDMA (or PVDMA_FL_; 20 mM) were prepared in acetonitrile and used for layer-by-layer assembly on SiO_2_ templates (average diameter = 4.86 μm). Empty microcapsules functionalized with DMAPA were fabricated by repeating the procedure described above.

### Encapsulation of Anisotropic and Isotropic Oils in Polymer Microcapsules

Empty capsules were rinsed and washed with ethanol twice and centrifuged down into a pellet. Droplets of LCs were encapsulated in the microcapsules using a previously described procedure.^28–31^ Briefly, the pellet in the microcentrifuge tube was suspended in 10 μL of ethanol (or a 0.1 mg/mL ethanol solution of Nile Red for caged droplets used for confocal microscopy, see text) and shaken gently. E7 (55 μL) was then added and the resulting mixture was placed on an automated shaker plate at room temperature for 15 h in a closed container. After 15 h, the microcentrifuge tube was uncapped and left open for 24 h to allow the ethanol to evaporate. Over this time period, E7 was observed to reform a nematic phase, resulting in the trapping of LC in the microcapsules. Excess E7 was removed by centrifugation and the remaining LC-filled capsules were extracted into DI water with gentle shaking. For other experiments using the model isotropic oil C14, the pellet of empty capsules was suspended in 10 μL of acetone, and C14 (40 μL) was then added and the process described above was repeated to prepare C14-filled microcapsules suspended in an aqueous phase. Reactive hydrophobic monomers were encapsulated by suspending the pellets of empty capsules in a solution of DMPAP (3 mg DMPAP dissolved in 12 μL ethanol, see text), followed by the addition of benzyl methacrylate (45 μL) and 1,6-hexanediol dimethacrylate (15 μL). The process described above was then repeated to extract the capsules filled with reactive monomers (referred to as BzMA-filled capsules) into an aqueous phase.

### Characterization of Oil-Filled Microcapsules

A 20 μL dispersion of oil-filled capsules was placed onto a glass coverslip and the capsules were allowed to settle and attach loosely through physical interactions to the surface for ∼15 min in order to facilitate characterization by microscopy. Experiments to investigate dynamic changes in the shapes of LC-filled capsules in response to surfactants were performed by adding concentrated solutions of SDS and DTAB to surface immobilized capsules to achieve desired final concentrations, and changes in the shapes and optical appearances of the LC-filled capsules were recorded using video microscopy. Experiments to investigate the dependence of capsule shapes on SDS concentration were performed by adding concentrated solutions of SDS (45 μL) to the dispersion of capsules (15 μL) to achieve desired final concentrations. Bright-field images of LC-filled capsules were acquired after 30 min of incubation in solutions of SDS. Changes in the shapes of LC-filled capsules over longer time periods (e.g., hours or days, see text) were examined by sampling dispersions of the incubated capsules in the presence of SDS at different time points. C14- and BzMA-filled capsules were characterized using the same general procedures. All experiments were performed at room temperature unless otherwise noted.

### Polymerization of Caged Oil Droplets and Characterization of Polymer Particles

A dispersion of BzMA-filled capsules (150 μL) was dispensed into a confocal dish and covered by a round glass coverslip. The BzMA-filled capsules were cured in an XL-1500 UV Cross-linker (Spectronics Corporation) using 365 nm UV illumination for 10 min, yielding solid polymer particles inside the capsules. Unreacted monomer was removed by rinsing with ethanol. The capsules were then resuspended in DI water by sonication, immobilized onto glass coverslips as described above, and characterized using bright-field microscopy. For experiments designed to prepare bare polymer particles without capsules, degradable microcapsules fabricated using PVDMA and cystamine (see above) were used.^33^ The oil droplets in these capsules were polymerized using UV light as described above and resuspended in aqueous solutions of DTT (10 mM) three times (10 min for each exposure) to remove the multilayer capsules. The resulting bare polymer particles were washed three times with DI water and characterized using bright-field microscopy. Translation and rotation of polymerized particles in aqueous media resulting from Brownian motion was recorded by video microscopy. For ease of characterization, a few drops of glycerol were added to the dispersion of particles to increase the viscosity of the aqueous phase. Samples for SEM imaging were prepared by placing a 1 μL dispersion of polymerized particles (with or without surrounding capsules) onto a silicon chip, followed by removal of water under vacuum and subsequently coating with a thin layer of gold.

## Results and Discussion

We reported previously that hollow and semipermeable polymer capsules fabricated by the layer-by-layer assembly of PVDMA and PEI can be infused with the nematic and water-immiscible LC E7 (an anisotropic oil) and then extracted into water to yield polymer capsules that are partially filled with small LC droplets.^28–30^ This overall process and the general structure of these “caged” LC droplets is shown schematically in Figure 1A,B (additional details related to the fabrication of PEI/PVDMA capsules and the infusion of LC are included in the Materials and Methods section, and additional discussion can also be found in several past publications^28–30^ ). The partially filled caged oil droplet morphology of these materials (Figure 1B) is understood to arise from the swelling of the positively charged, hydrophilic PEI/PVDMA capsules once they are extracted into aqueous media and, as demonstrated below, is generalizable to the design of partially filled capsules using a variety of other types of hydrophobic liquids, including simple hydrocarbon oils and reactive hydrophobic liquid monomers (e.g., as shown schematically in Figure 1C).^31^

**Figure 1:**
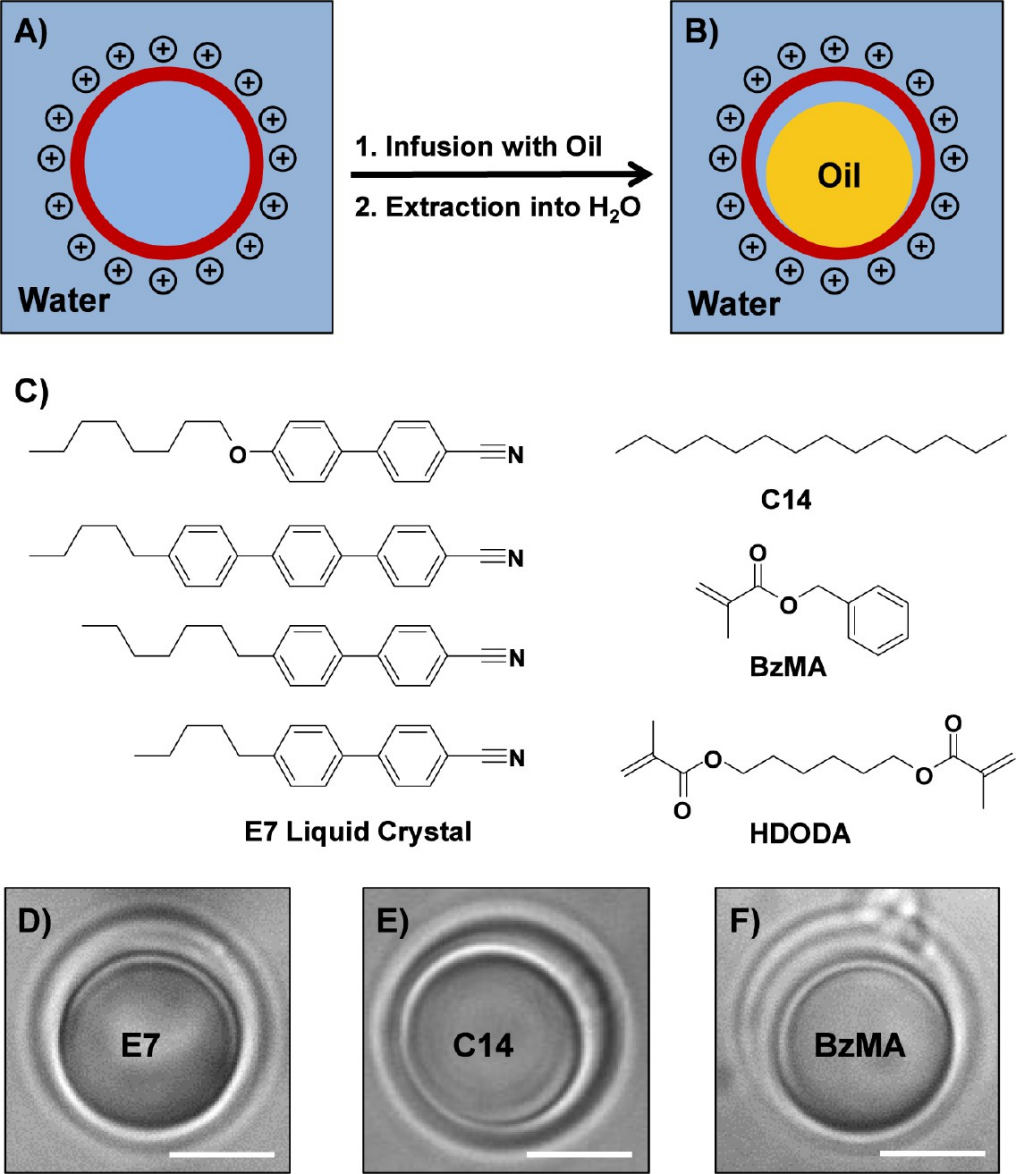
(A,B) Schematic illustration showing a hollow polymer capsule (A) and a hollow capsule partially filled with a smaller droplet of oil, yielding a “caged” oil droplet suspended in water (B). (C) Chemical structures of the hydrophobic molecules used in this study, including the thermotropic LC E7, the isotropic hydrocarbon oil tetradecane (C14), and the polymerizable oil benzyl methacrylate (BzMA) doped with the cross-linker 1,6-hexanediol dimethacrylate (HDODA) (D–F). Bright-field microscopy images of (D) a caged E7 droplet, (E) a caged C14 droplet, and (F) a caged BzMA droplet suspended in water. Scale bars are 5 μm.

Our past studies were performed using caged LC droplets prepared using hollow PEI/PVDMA capsules ∼5 μm in size. This current study sought to investigate the influence of the size and thickness of the polymer “cages” on the properties and behaviors of the “caged” oil droplets. For these experiments, we fabricated PEI/PVDMA capsules ∼8 μm in diameter (referred to from here on as “8 μm-large” capsules) that were larger than the ∼5 μm templates used in past studies^28–30^ (referred to from hereon as “5 μm-small” capsules) by using layer-by-layer assembly to deposit layers of PEI and PVDMA onto sacrificial spherical silica templates. This layer-by-layer process also provides a straightforward approach to varying the thicknesses of the polymer capsules by varying the number of PEI/PVDMA layer pairs (or “bilayers”) deposited during assembly. We measured the walls of PEI/PVDMA capsules four bilayers thick fabricated on “8 μm-large” spherical templates to be 54.9 ± 7.4 nm using atomic force microscopy (AFM; see Figure S1), and past studies reveal these reactive multilayers to increase, in general, in a manner that is linear with respect to the number of polymer layers added.^34^ Additional measurement of the shear modulus of these hollow capsules using a previously reported osmotic buckling method^35^ revealed the average shear modulus when suspended in water to be approximately 38 MPa (see Figure S2).

In a first series of experiments described in the first section of this paper, we infused the nematic LC E7 into “8 μm-large” PEI/PVDMA capsules 4.5 bilayers thick to yield partially filled, spherical caged LC droplets suspended in deionized water (Figure 1D). We later expanded this approach to the fabrication of caged isotropic oil droplets, including a nonreactive hydrocarbon oil and a mixture of photopolymerizable monomers (Figure 1E,F), and to thicker cages fabricated by the deposition of 6.5 bilayers of PEI and PVDMA (described in greater detail in subsequent sections). These “8 μm-large” caged oil droplets exhibited a small spherical droplet of oil (∼8.0 μm in diameter, defined by the size of the original size of the capsules during infusion with oil) contained within a larger spherical shell (∼11.0 μm in diameter, larger than the original size of the capsules as a result of swelling in the aqueous phase, as noted above). Overall, these morphologies were generally similar to those observed for partially filled “5 μm-small” caged LC droplets reported in past studies.^28–30^

### Characterization of Changes in the Shapes of Caged LC Droplets upon Exposure to Surfactants

Past studies demonstrate that spherical (or nearly spherical) LC droplets caged in “5 μm-small” capsules can undergo changes in shape, mobility, and contact angles upon exposure to model surfactants.^28–30^ For example, addition of a model anionic surfactant, sodium dodecyl sulfate (SDS), can result in a continuous decrease in droplet/capsule contact angles, yielding droplets with a series of lens-like shapes [from convex lens-like shapes at low concentrations of SDS (0.05 to 0.25 mM) to concave lens-like shapes at moderate concentrations of SDS (0.5 to 1.0 mM); the critical micelle concentration (CMC) of SDS is 8.2 mM in pure water at 25 °C] contained within polymer capsules that do not undergo substantial deviations from their initial spherical shapes.^29,30^ In light of these past results, we performed a series of experiments to investigate the responses of droplets encapsulated in spherical 8 μm-large capsules 4.5 bilayers thick to the addition of different concentrations of SDS.

In sharp contrast to wetting transitions observed in smaller caged LC droplets described above, droplets contained in these 8 μm-large capsules were observed to undergo large transformations in shape upon the addition of very low concentrations of SDS (1 μM; ∼10^–4^ of the CMC). Figure 2 (top row, A–E) shows representative bright-field micrographs of different morphologies of caged LC droplets observed in 1 μM SDS, including a mixture of roughly spherical caged droplets and droplets that exhibit complex anisotropic shapes and apparent 6-, 5-, 4-, and 3-fold symmetries. To provide additional insights into the morphologies of these droplets, we characterized caged LC droplets doped with Nile Red, a hydrophobic fluorescent dye, in the presence of 1 μM SDS using confocal microscopy [the capsules used in this experiment were also fabricated using fluorescently labeled PVDMA (PVDMA_FL_); see Materials and Methods]. Inspection of the representative 2D projections of these capsules in Figure S3 and the corresponding 3D reconstruction images in Figure 2 (bottom row, F–J) reveals several key observations. First, the apparently spherical caged droplets observed using bright-field microscopy (Figure 2A) indeed consist of spherical polymer shells (green, Figure 2F) and roughly spherical LC droplets (red, Figure 2F). In contrast, the caged droplets with complex shapes appear to contain nonspherical LC droplets with apparent 6-, 5-, 4- and 3-fold symmetries (Figure 2B–E,G–J). The polymer shells surrounding these deformed droplets were in most cases not completely deformed; in general, some parts of the capsules appeared to be in contact with the droplets, and other regions of the capsules did not. These results, when combined, reveal changes in shapes of both deformable polymer shells and encapsulated droplets that have not been observed and would likely be difficult to obtain by the addition of SDS to bare LC emulsion droplets^36,37^ or completely filled capsules.^38–40^ (We note that the focus of the work reported here is on understanding factors underlying these large changes in shape and not on the optical properties of these anisotropically shaped LC droplets; characterization of differences in the director profiles and optical properties of the LCs that arise from these shapes will be described in a separate report. We note further that while several past studies have reported the design of hollow and stimuli-responsive nonspherical capsules by the deposition of polymer multilayers on the surfaces of sacrificial nonspherical templates,^41–45^ the structures and behaviors of the oil-filled capsules reported here and discussed below differ substantially from that past work).

**Figure 2:**
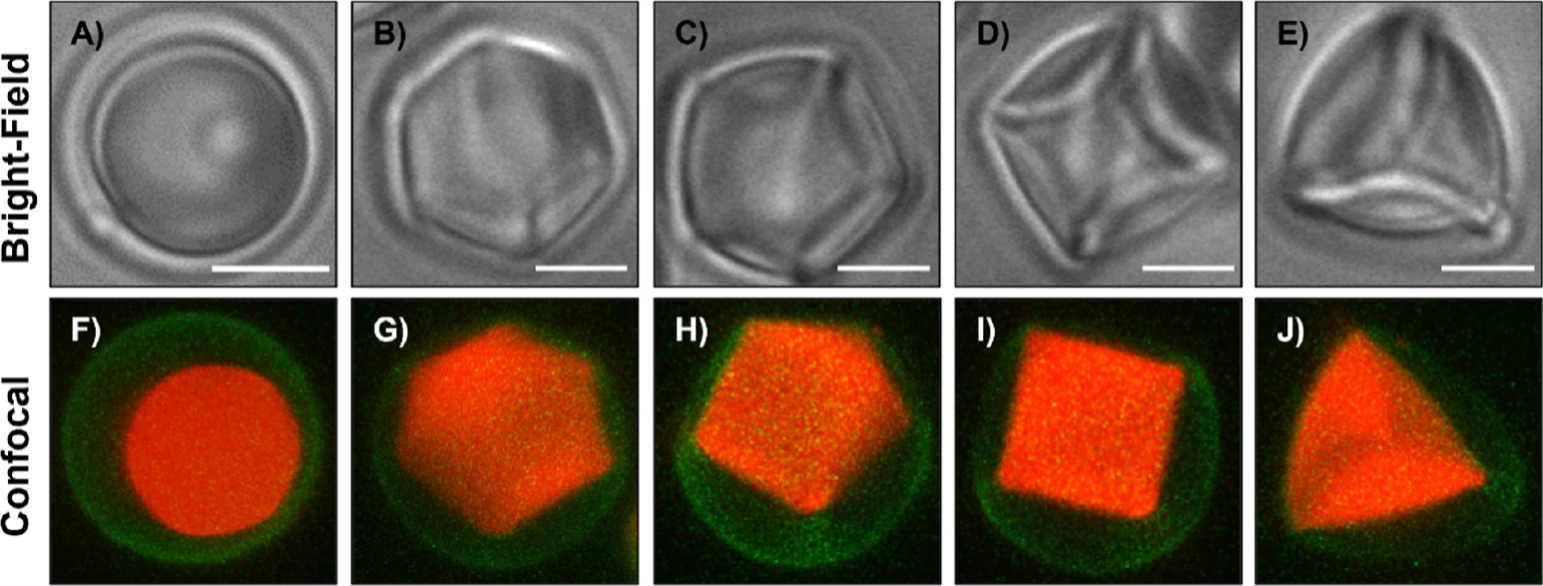
Bright-field microscopy images (top row; A–E) and 3D reconstruction confocal microscopy images (bottom row; F–J) of caged LC droplets suspended in 1 μM SDS. The images show the variety of deformations and shapes with apparent 6-, 5-, 4-, and 3-fold symmetries observed (text). Scale bars are 5 μm.

Past studies demonstrate that changes in the contact angles of “5 μm-small” caged LC droplets depend largely on concentrations of added surfactants.^28–30^ To determine the extent to which the stimuli-responsive shape changes and the distributions of colloidal droplet shapes described above were dependent on the amount of SDS present in solution, we characterized 4.5-bilayer, “8 μm-large” caged LC droplets upon exposure to increasing concentrations of SDS (Figure 3; a complete data set showing capsules at various concentrations of SDS is shown in Figures S4 and S5). For these experiments, water or serial solutions of SDS were added to aqueous suspensions of caged LC droplets to achieve desired final concentrations. The results in Figure 3 (and in Figures S4 and S5) reveal large changes in colloidal droplet shapes and the distributions of different shapes as a function of SDS concentration. As shown in Figure 3A,B, the majority of caged LC droplets remained spherical at 0 μM SDS. As concentrations of SDS increased, caged droplets with folded polymer shells and anisotropic shapes started to emerge (Figures S4A–C and S5A–C). At 1 μM SDS, a mixture of caged LC droplets with apparent 6-, 5-, 4-, and 3-fold symmetries was observed (Figure 3C,D). Continuous increases in the concentration of SDS led to increases in populations of droplets with lower order symmetries (e.g., 4- and 3-fold symmetries), until droplets with 3-fold symmetries became the dominant morphology (at 10 μM SDS; the population of droplets with 3-fold symmetries was ∼73% of the total population; Figures 3E,F, S4D–G and S5D–G). The distribution of droplet shapes was not observed to change substantially with further increases in SDS concentration (from 10 μM to 1 mM; Figures S4G–I, and S5G,H). These results, when combined, suggest the relative stability of droplets with 3-fold symmetries as compared to droplets with other shapes. Additional experiments revealed that the shape distribution of droplets in solutions of 1 μM SDS did not change substantially after 3 days of incubation at room temperature (Figure S6C).

**Figure 3:**
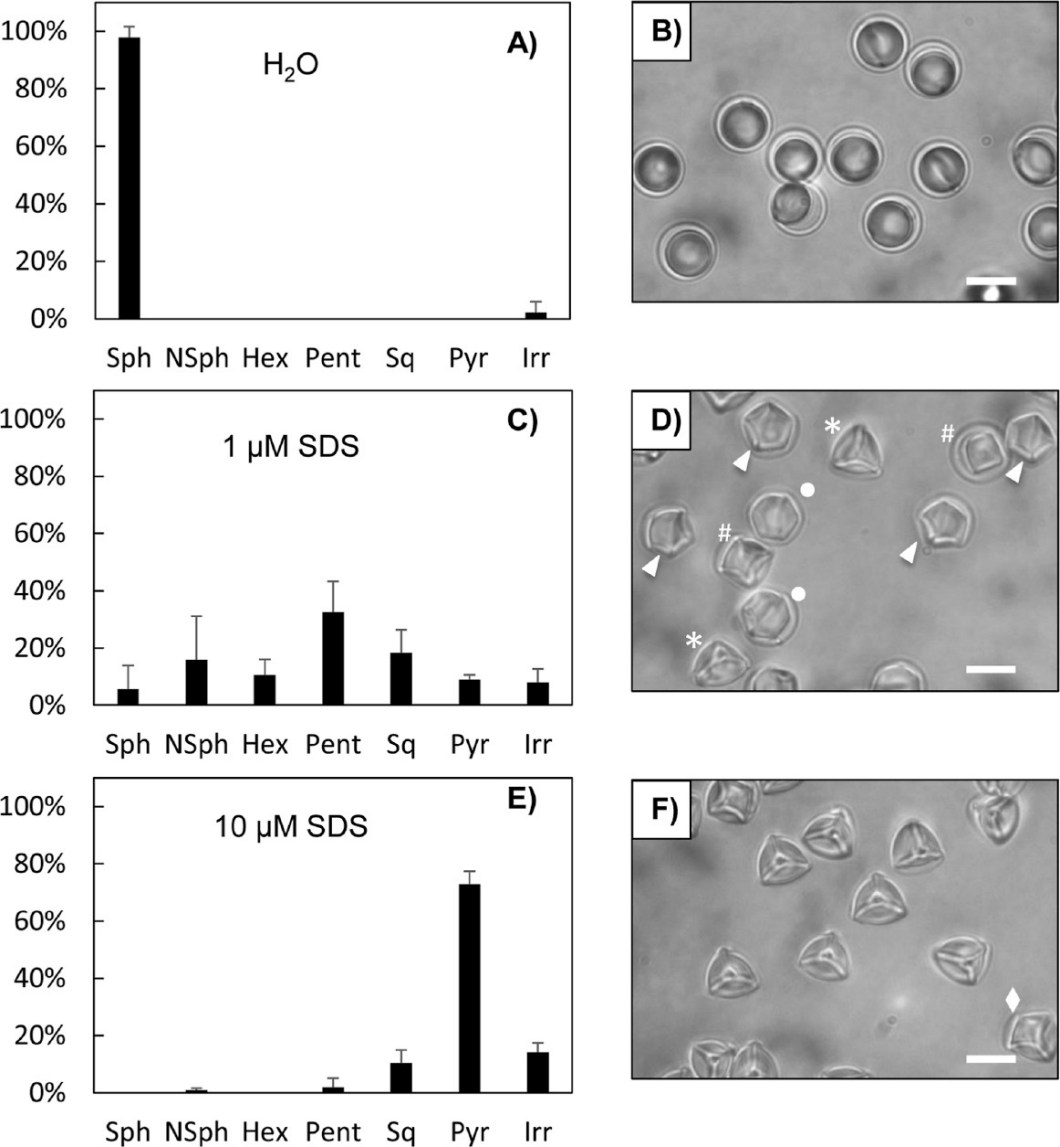
Plots and corresponding bright-field microscopy images showing distributions of caged LC droplets with different apparent shapes in (A,B) 0 μM SDS, (C,D) 1 μM SDS, and (E,F) 10 μM SDS; Sph = spherical; NSph = nonspherical; Hex = hexagon; Pent = pentagon; Sq = square; Pyr = pyramid; Irr = irregular shaped. Caged droplets with 6-fold (white dots), 5-fold (arrowheads), 4-fold (pound signs), and 3-fold (asterisks) symmetries, and other intermediate or irregular shapes (diamond) were observed. The number of caged droplets measured was *N* > 150 for each plot. Error bars are standard deviations of three parallel samples. Scale bars are 10 μm.

We further monitored time-dependent changes in droplet shapes upon exposure to SDS using video microscopy. For these experiments, free-floating spherical 4.5-bilayer, “8 μm-large” caged LC droplets suspended in deionized water were allowed to settle and attach onto negatively charged bare glass coverslips through electrostatic interactions to facilitate the characterization of individual particles over time. A droplet of a concentrated SDS solution was added to these surface-immobilized capsules to achieve a final concentration of 10 μM. Figure 4 (top) shows a series of real-time snapshots of a video recorded after the introduction of SDS to a spherical caged LC droplet. Inspection of these snapshots revealed that the droplet lost symmetry almost instantaneously upon exposure to SDS, transforming from the initial axisymmetric spherical shape to a curvilinear polyhedral shape, and evolved continuously from shapes with higher orders of symmetry to shapes with lower orders of symmetry over a period of 95 s. This process, accompanied by a reduction in the symmetry of apparent capsule folds and an apparent increase in capsule/droplet contact area, ultimately led to the formation of a droplet/capsule structure having a 3-fold symmetry. The morphology of the droplet did not change thereafter for up to ∼45 min. Changes were also not observed for droplets with this 3-fold symmetry incubated in 10 μM SDS solutions over several days, consistent with the observations described above. These results further suggest that droplets with this 3-fold symmetry are more stable than droplets with other higher-order anisotropic shapes.

**Figure 4:**
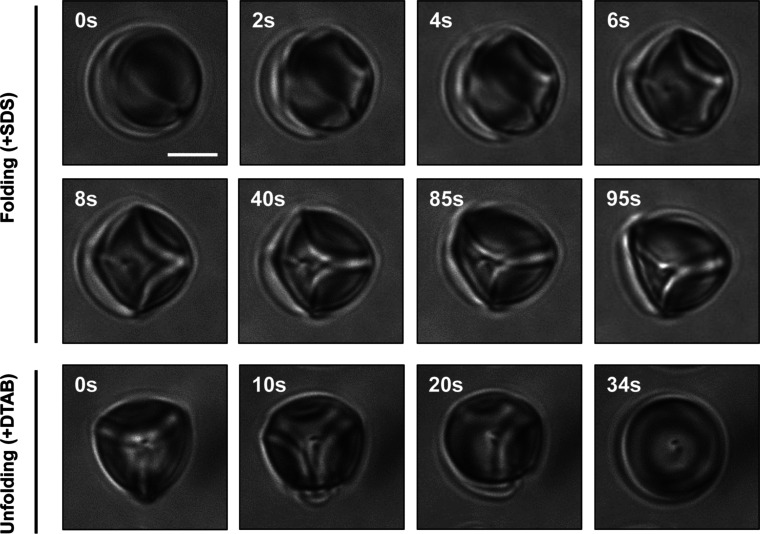
Representative bright-field microscopy images of (top) a caged LC droplet at various times after the introduction of 10 μM SDS and (bottom) a droplet/capsule structure with an initial 3-fold symmetry suspended in water at various times after the introduction of 1 mM DTAB. Scale bars are 5 μm.

The time scale for a spherical caged droplet to fold or buckle into a droplet/capsule structure with the 3-fold symmetry exhibited here upon exposure to SDS ranged from several seconds to approximately 2 min. Some capsules did not transform completely into structures with 3-fold symmetry during the period of our observation but appeared to be kinetically trapped in intermediate states (e.g., the droplet labeled with the diamond in Figure 3F; see additional discussion below). Similar symmetry breaking behavior has been observed during the buckling of elastic shells.^46–51^ Deformation of macro/microscopic spherical elastic shells under external pressure (e.g., osmotic pressure or point indentation) beyond a critical volume results in the symmetry breaking of dimples from higher order to lower order nonaxisymmetric shapes, a phenomenon known as the “secondary-buckling” of elastic shells.^46–51^ However, hollow 4.5-bilayer “8 μm-large” PEI/PVDMA capsules free of oil droplets were not observed to undergo changes in shapes upon exposure to SDS (Figure S7), suggesting that the SDS-induced shape changes observed here are more likely to arise from changes in interactions between encapsulated LC droplets and their surrounding polymer capsules than from external pressures.

Additional experiments revealed that droplet/capsule structures with 3-fold symmetry remained in this state for at least 30 min after replacing SDS solutions with deionized water (Figure S8). However, they were observed to return to their original spherical shapes rapidly (in less than 1 min) upon the addition of a model cationic surfactant, decyltrimethylammonium bromide (DTAB). As shown in Figure 4 (bottom), a droplet/capsule structure with 3-fold symmetry in water was exposed to 1 mM DTAB. Both the capsule wall and the encapsulated droplet were observed to return to spherical shapes in ∼34 s. These results, in general, are consistent with observations of different wetting transitions in LC droplets caged in “5 μm-small” capsules caused by surfactants with different head groups or charges.^29,30^ Past studies of these smaller caged LC droplets have demonstrated that addition of DTAB can induce an increase in droplet/capsule contact angles (i.e., dewetting of the encapsulated droplets), in contrast to wetting transitions caused by the addition of SDS.^29,30^

### Discussion of Possible Mechanisms for Changes in the Shapes of Caged LC Droplets

On the basis of the results described above and those reported in past studies, we propose a possible explanation for the SDS-induced changes in the shapes of caged LC droplets observed here based on the minimization of the total free energy *F*_total_ of the system (a combination of total surface free energy, *F*_surface_, and the elastic energy of the capsule wall, *F*_elastic_; other contributions to *F*_total_ are neglected; eq 1).

1 $$F_{total}=F_{surface}+F_{elastic}$$

In the initial state, a caged LC droplet suspended in deionized water is observed to be in a spherical shape (as shown schematically in Figure 5A). We define the interfacial areas between the LC and the aqueous phase, the LC and the capsule wall, and the capsule wall and the aqueous phase as *A*_Oil‑Aq_, *A*_S‑Oil_, and *A*_S‑Aq_, respectively, and the corresponding interfacial tensions as γ_Oil‑Aq_, γ_S‑Oil_, and γ_S‑Aq_. Thus, the total surface free energy, *F*_surface_, can be written as

2 $${F_{surface}=\gamma }_{S‐Aq}A_{S‐Aq}+\gamma_{Oil‐Aq}A_{Oil‐Aq}+\gamma_{S‐Oil}A_{S‐Oil}$$

**Figure 5:**
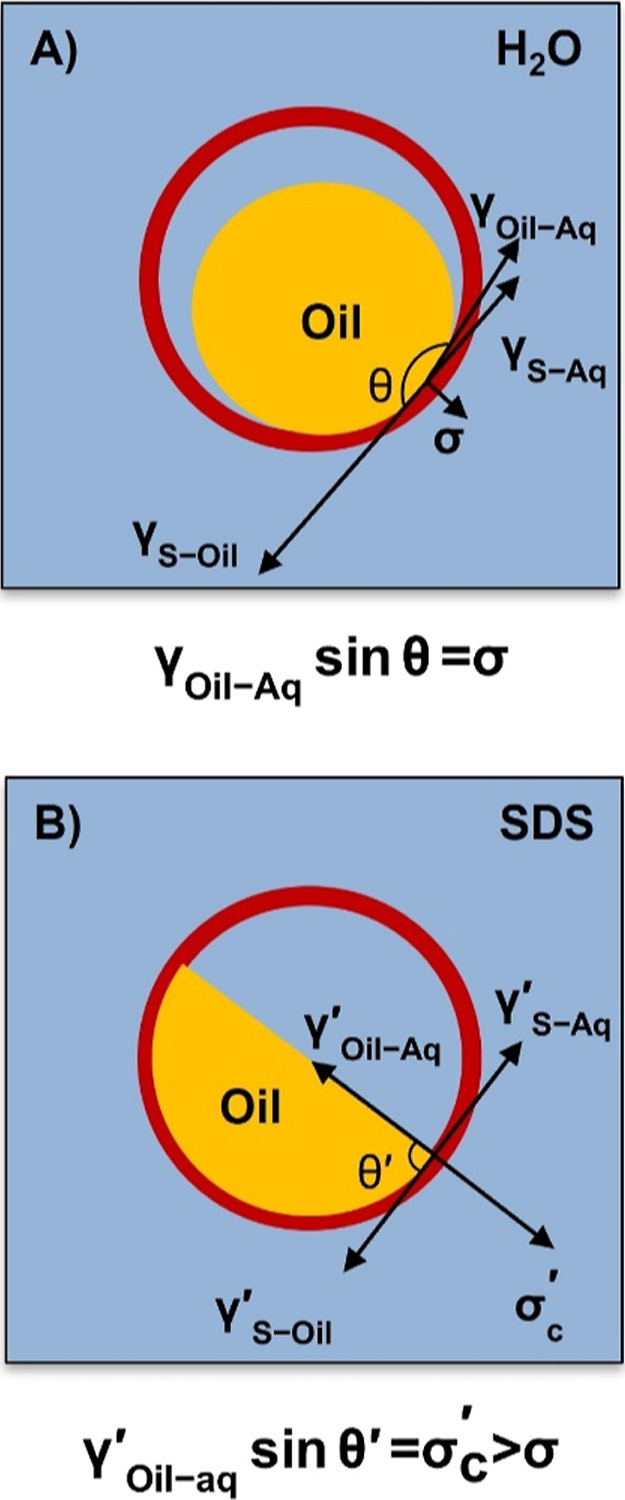
Schematic illustration showing force balances in caged oil droplets (A) before and (B) after adding SDS.

We note that the three-phase contact angle observed in the initial state is high (θ ∼ 160°), resulting from the hydrophobic nature of the LC droplet and the hydrophilic nature of the presumably positively charged wall of the polyamine-based capsule. Assuming that the morphology of the caged LC droplet observed here represents an equilibrium state, it is possible to formulate force balance equations at the three-phase contact line along tangential (eq 3; Young’s Equation) and normal directions (eq 4) to the capsule wall

3 $$0=\gamma_{S‐Aq}-\gamma_{Oil‐Aq}\cos\theta -\gamma_{S‐Oil}$$

4 $$0=\gamma_{Oil‐Aq}\sin\theta -\sigma_{S}$$

The large contact angle also serves to reduce interactions between the encapsulated LC droplet and the capsule wall suggesting, in view of eq 3, that γ_S‑Oil_ is dominant over γ_Oil‑Aq_ and γ_S‑Aq_ in the initial state and the normal force, σ_S_, is small (eq 4).

Past studies have demonstrated that the contact angle, θ, decreases when the “5 μm-small” caged LC droplets are exposed to SDS (as shown schematically in Figure 5B).^29^ These results suggest changes in interfacial tensions (i.e., decreases in γ_S‑Oil_ or increases in γ_S‑Aq_ and/or γ_Oil‑Aq_) resulting from the adsorption of SDS to *A*_S‑Oil_, *A*_S‑Aq_ and/or *A*_Oil‑Aq_. In view of these results, and our observations of the increase in droplet/capsule contact areas during shape change, we reasoned that for “8 μm-large” caged LC droplets, addition of low concentrations of SDS could result in a significant decrease in the originally dominant γ_S‑Oil_ by adsorption of anionic SDS to the inner surface of the cationic capsule wall and transform it into a more hydrophobic surface. As a result, a droplet would tend to wet the inner surface of the capsule wall and exhibit a lower contact angle, θ′. Such changes would result in a significant increase in the normal force, σ_S_′ as the interfacial tension, γ_Oil‑Aq_′, should not change substantially at low SDS concentration (we note that if γ_Oil‑Aq_′ did increase in magnitude, as discussed above, this would also increase the normal force).^29,52^ If the polymer shell is “soft” enough (i.e., the elastic energy cost is small compared to the interfacial energy penalty), it should deform under this increased line tension to reduce the LC/aqueous phase interfacial area, *A*_Oil‑Aq_′, and, thus, the dominant high surface energy, γ_Oil‑Aq_′*A*_Oil‑Aq_′.^13^ In this way, the system can minimize the total free energy, *F*_total_, by reducing *F*_surface_ at the cost of increasing the elastic energy, *F*_elastic_.

Both bending and stretching deformations of the capsule wall are expected to be involved in the formation of the apparent folds described above.^46–51^ Bending energies for thin capsules generally scale like the cubed thickness, while stretching energies scale with the thickness, so capsule bending is generally a softer mode of deformation.^53^ However, focused regions of stretching are still expected where the curvature changes rapidly, as required by Gauss’s Theorema Egregium, which states that Gaussian curvature is invariant under stretch-free, smooth deformations.^54^ Related works on spherical capsules with compositions similar to the ones used here confirm that the elastic energy increases as the shell thickness or curvature increases,^46–51^ i.e., shells with thicker walls or smaller sizes are more difficult to deform due to high elastic energy penalties. In view of those reports, we investigated the influence of capsule size and shell thickness on SDS-induced shape changes. For these experiments, we fabricated additional 8 μm-large and 5 μm-small capsules that were 6.5 bilayers thick and loaded them with LC droplets as described above. Inspection of Figure 6 reveals that upon exposure to 10 μM SDS, only 4.5 bilayer capsules templated on 8 μm-large silica cores fold or buckle into droplet/capsule structures with 3-fold symmetries (Figure 6A). Other caged LC droplets with thicker walls and/or smaller sizes demonstrate wetting transitions of encapsulated LC droplets, but the capsule walls remained spherical in the presence of SDS (Figures 6B–D, and S9). These results are consistent with an increased elastic energy penalty associated with capsule deformation, as discussed above, and provide additional support for our proposed free energy formulation. In addition, the observed shapes are reminiscent of well-studied elastic shells and capsules collapsed by pressure differences^55–57^ and by other means.^58–60^ Although such deformations may be templated by surface inhomogeneities,^50,56,61,62^ in the present case the initial position of the internal oil droplet provides a natural localization of the surface collapse.

**Figure 6:**
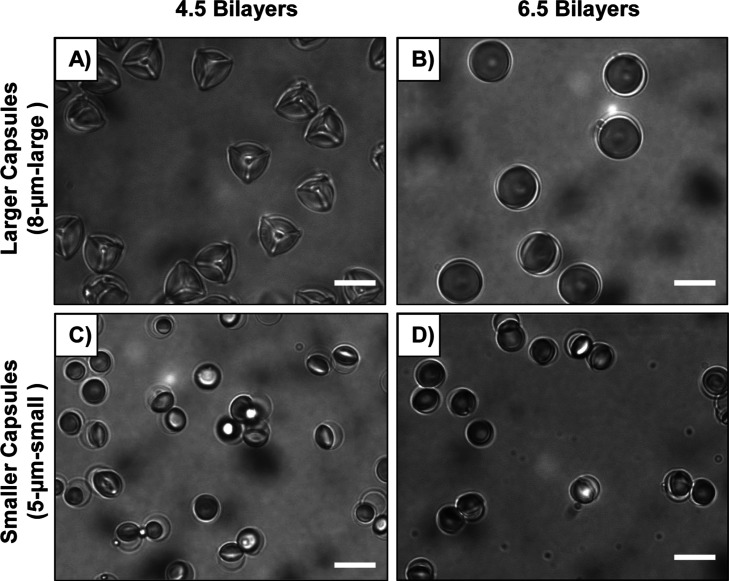
Bright-field microcopy images showing caged LC droplets with different sizes and thicknesses of the polymer “cages” suspended in 10 μM SDS (see text). Droplets caged in (A) 4.5 bilayer, “8 μm-large” capsules, (B) 6.5 bilayer, “8 μm-large” capsules, (C) 4.5 bilayer, “5 μm-small” capsules, and (D) 6.5 bilayer, “5 μm-small” capsules. Scale bars are 10 μm.

### Characterization of Changes in the Shapes of Caged Isotropic Oil Droplets

The simple free energy proposal described above only accounts for contributions from interfacial energies and the elastic energy of the capsule wall but does not consider any special properties of nematic LCs, such as the bulk elasticity of the LC droplets. However, the results discussed above suggest that this simple formulation is sufficient to qualitatively describe the SDS-induced shape changes observed in caged LC droplets, and that the nematic ordering of LCs does not appear to play a role in promoting these SDS-induced shape changes. Indeed, the results of additional experiments suggest that the shape changes observed in caged LC droplets do not arise from nematic ordering of the LC, and that this behavior is generalizable for capsules partially filled with other hydrophobic isotropic oils. To explore the generality of this stimuli-responsive shape change behaviors of caged oil droplets, we loaded tetradecane, a simple isotropic oil, into 4.5-bilayer “8 μm-large” PEI/PVDMA capsules to fabricate spherical caged isotropic oil droplets (Figure 1E). Upon exposure to 1 μM SDS, these spherical caged oil droplets were observed to undergo large transformations in shape in ways similar to those observed in caged LC droplets. Inspection of Column 1 of Figure 7 reveals folded caged droplets with complex shapes and apparent 6-, 5-, 4- and 3-fold symmetries similar to those shown in Figure 2.

**Figure 7:**
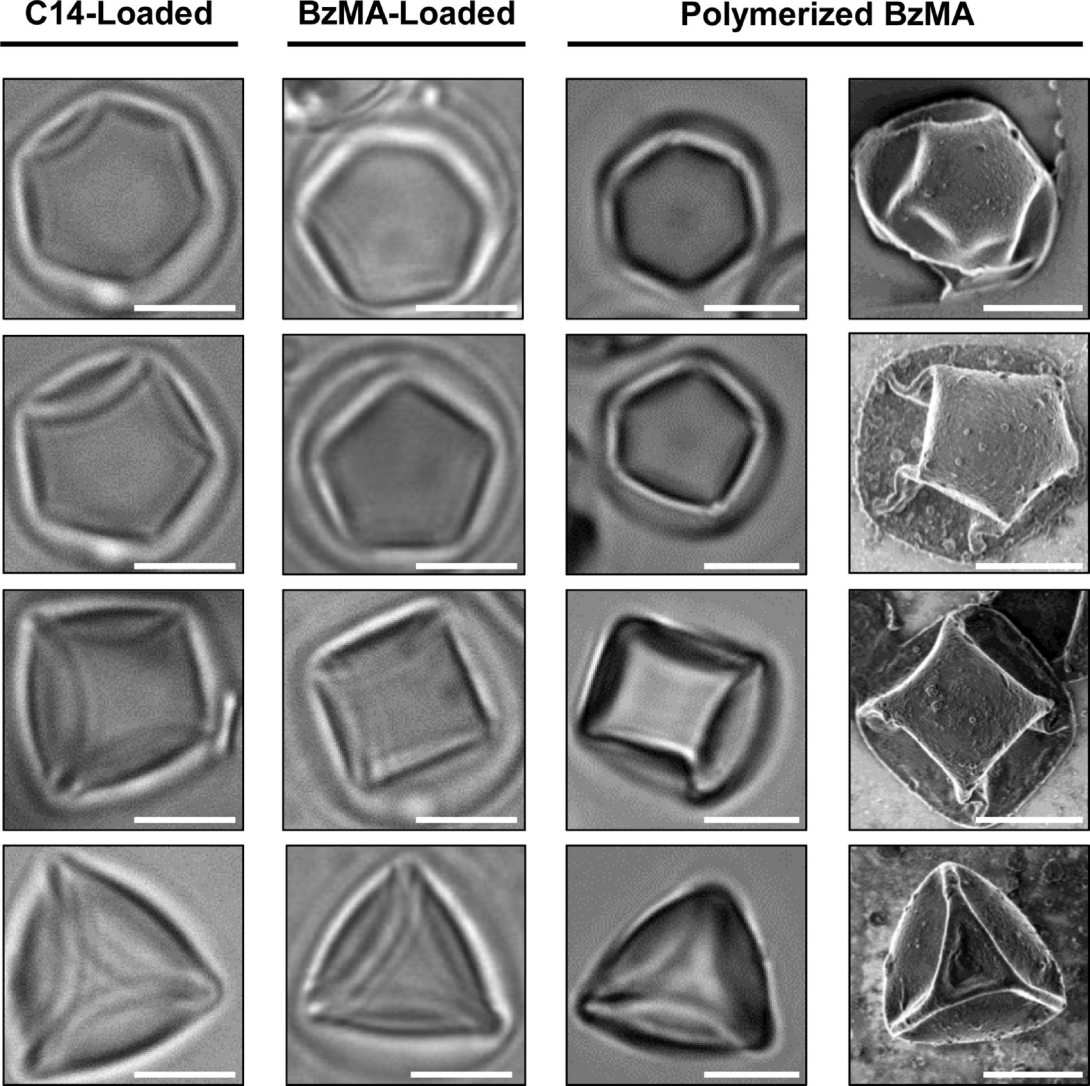
Bright-field microcopy images showing caged isotropic oils suspended in SDS: (column 1) caged C14 droplets in 1 μM SDS and (column 2) caged BzMA droplets in 20 μM SDS. Bright-field (column 3) and SEM (column 4) images of caged BzMA particles after polymerization in 20 μM SDS. Scale bars are 5 μm.

We further demonstrated that this approach could also be coupled with the use of a mixture of photopolymerizable hydrophobic oils consisting of an acrylate monomer, benzyl methacrylate (BzMA, 75% (w/w)), and a diacrylate cross-linker, 1,6-hexanediol dimethycrylate (HDODA, 25% (w/w)) (Figure 1C). For these droplets, addition of SDS also yielded caged reactive oil droplets with anisotropic shapes similar to those described above (Column 2 of Figure 7). Subsequent photopolymerization of these caged reactive oil droplets dispersed in SDS yielded solid polymer particles caged inside polymer capsules (Column 3 of Figure 7). The encapsulated polymer particles were observed to preserve the anisotropic shapes of the liquid droplets prior to polymerization. Characterization of these caged polymerized particles using SEM revealed that they were covered with folded, deformed polymer capsules (Column 4 of Figure 7). Because these thin polymer capsules conformed tightly to the surfaces of the polymer particles inside, these SEM images also revealed information about the shapes and morphologies of the encapsulated particles. As shown in Column 4 of Figure 7, caged polymer particles with anisotropic shapes and 6-, 5-, 4-, and 3-fold symmetries were observed, consistent with our observations of these particles using optical microscopy. These results, when combined, demonstrate that the shapes of the reactive oil droplets can be “locked” in situ by photopolymerization, providing a strategy for the synthesis of caged polymer particles with anisotropic shapes templated on the original buckled or deformed oil-filled capsules.

### Fabrication and Characterization of “Cage-Free” Polymer Particles with Anisotropic Shapes

To determine whether the approach described above could be used for the synthesis of “cage-free” anisotropic polymer particles without surrounding shells, we loaded polymerizable oils into reductively degradable capsules fabricated by the reactive layer-by-layer assembly of PVDMA and a disulfide-containing linker (see Materials and Methods for additional details about this fabrication process).^31^ This approach led to spherical, degradable polymer shells that were partially filled with smaller spherical droplets of reactive oils (Figure S10A). Upon exposure to SDS, the droplets in these reductively degradable shells were also observed to undergo changes in shape, yielding caged droplets with 6-, 5-, 4- and 3-fold symmetries similar to those observed in PEI/PVDMA capsules (Figure S10B). Photopolymerization of these droplets, followed by removal of the degradable polymer shells by reduction with DTT, yielded “cage-free” solid polymer particles with shapes that were templated by those of the original caged droplets prior to polymerization (Figure 8A). Figure 8A shows a representative optical microscopy image of polymer particles resulting from these experiments and reveals a mixture of bare polymer particles with different shapes, including anisotropic shapes with apparent 6-, 5-, 4-, and 3-fold symmetries and other irregular shapes that could arise from droplets trapped in intermediate states or from side-on or oblique views of particles with the more regular symmetries described above.

**Figure 8:**
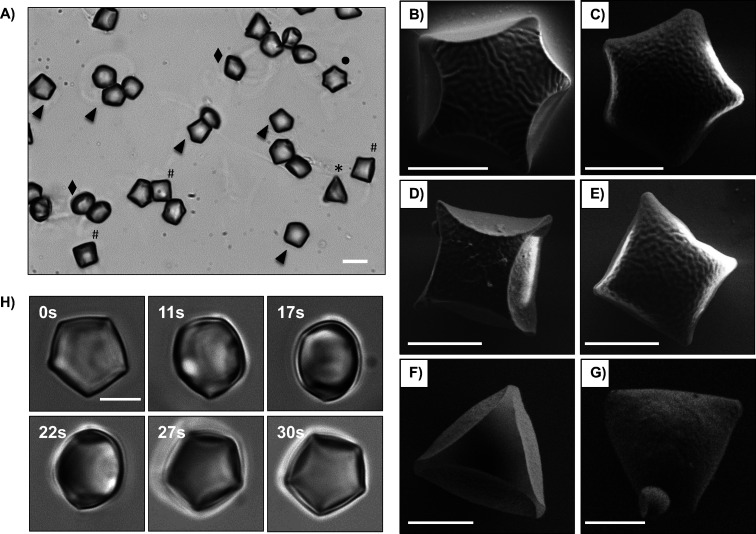
Bright-field microscopy images (A) and SEM images (B–G) showing “cage-free” polymerized particles after treatment with 10 mM DTT. Polymer particles with apparent 6-fold (dot in A), 5-fold (arrowheads in A; B,C), 4-fold (pound signs in A; D–E), and 3-fold (asterisk in A; F,G) symmetries, and other intermediate or irregular shapes (diamonds) were observed. (H) Representative bright-field microscopy images of a “cage-free” polymer particle with 5-fold symmetry rotating and translating in aqueous solution due to Brownian motion. Scale bars are 10 μm (A) and 5 μm (B–H).

To provide additional insights into the shapes and surface morphologies of these “cage-free” polymer particles, we characterized the particles using SEM. As shown in Figures 8B–G and S11, the “cage-free” polymer particles exhibited anisotropic shapes that were similar to those of the “caged” particles observed in Column 4 of Figure 7. Residual polymer films and broken pieces of capsules were generally not observed in these images, suggesting complete removal of the reductively degradable polymer shells. These “cage-free” polymer particles appeared to have pore-free surfaces, with some surfaces having microscale wrinkles that could arise from shrinkage during polymerization. Further inspection of Figures 8B–G and S11 reveals additional details of particle morphologies that are not apparent from bright-field views or SEM images of caged particles. For example, the particle with 5-fold symmetry in Figure 8C demonstrates a rounded curvature with a smooth transition across the surface (referred to as a “bottom” surface; see discussions below); no evidence of a sharp boundary was observed. In contrast, the particle shown in Figure 8B, which also has an apparent 5-fold symmetry, was observed to exhibit a sharp edge with the same order of symmetry that clearly divided the surface of the particle into two distinct surface morphologies. The surface enclosed by the sharp edge, which we refer to as a “top” surface (see discussions below), exhibits 5-fold symmetry and also appears to be smaller than the base area of the particle. Particles with 6-, 4- and 3-fold symmetries were also observed to exhibit two types of surfaces in analogy to the “top” and “bottom” surfaces described above (Figures 8D–G and S11A). Additional video microscopy observations of a polymer particle with 5-fold symmetry tumbling in solution (Figure 8H) suggests that the two different surfaces observed by SEM represent “top” and “bottom” views of particles having this 5-fold symmetry. As shown in Figure 8H, the surface of the particle at *t* = 0 s appeared to be smooth, similar to that observed in Figure 8C, while the surface of the same particle at *t* = 30 s exhibited a surface with a shape, morphology, and sharp edges, similar to those observed in Figure 8B. The particle at intermediate times showed “side” views with irregular shapes. Similar results were also observed in particles with 6-, 4-, and 3-fold symmetries.

Observed morphologies of the “top” and “bottom” surfaces of the particle likely arise from a folded or buckled capsule/droplet structure having an overall shape, after the addition of SDS, such as the one represented in the cartoon shown in Figure 9B, in which the rounded “bottom” surface of the particle arises from the part of the liquid droplet that is wrapped conformally by the polymer capsule wall, and the “top” surface arises from the part of the droplet that is in contact with the aqueous phase (Figure 9B). The sharp edge with 5-fold symmetry described above (Figure 8B) likely represents the contour of the three-phase contact line, or the boundary of the water/oil/capsule wall interface (Figure 9B). The shape and the overall top-bottom anisotropy of the droplet was preserved during polymerization. Subsequent removal of the degradable capsule yielded the “cage-free” solid polymer particle represented in the cartoon in Figure 9A, which shows a particle with 5-fold symmetry, with its shape and morphology templated by that of the original liquid droplet.

**Figure 9:**
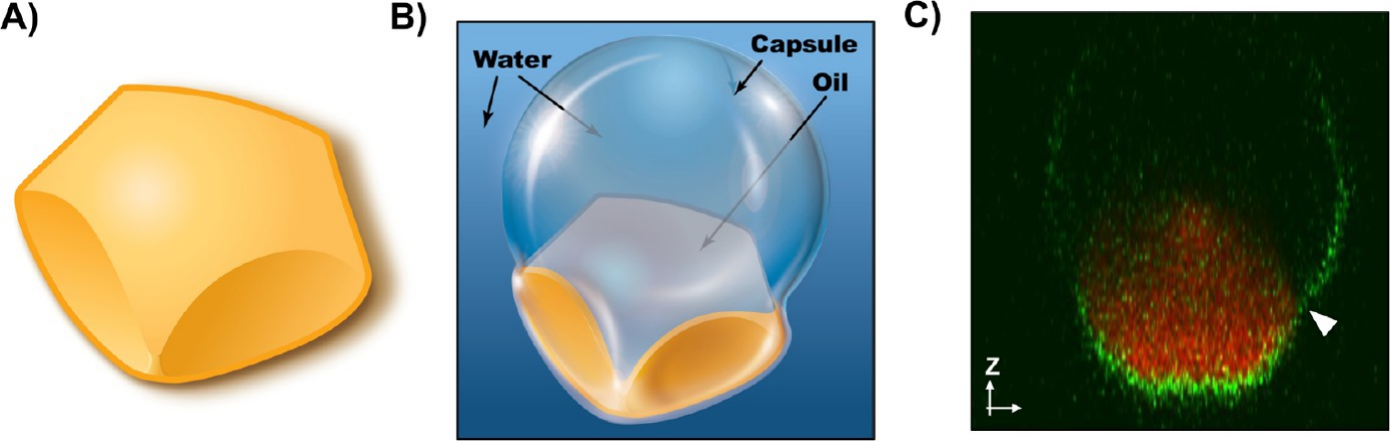
Schematic illustration presenting a physical picture of (A) a polymer particle with 5-fold symmetry and (B) a droplet/capsule structure with 5-fold symmetry. This view shows conformal contact of the capsule with two front-facing sides of the oil droplet. Experimental observations suggest that all the other three faces of this structure, which are hidden from view in this illustration, are otherwise similar. (C) 2D projection confocal image (along the *z* direction) of a caged LC droplet with 5-fold symmetry. The white arrowhead marks the boundary of the water/oil/capsule wall interface.

Additional evidence in support of the overall deformed oil droplet/capsule structure depicted in the cartoon in Figure 9B was provided by a 2D confocal microscopy projection (along the *z* direction) of a capsule/droplet structure with 5-fold symmetry (Figure 9C). Inspection of Figure 9C reveals that the part of the capsule wall (green) in immediate contact with the droplet (red) deformed to conform to the surface curvature of the droplet. The part of the capsule wall that was in contact with water appeared to remain roughly spherical (apparent elongation of the capsule in the *z*-direction is an optical artifact).^63^ There was also a clear boundary where the capsule wall deviated from the surface of the droplet (marked by the white arrowhead), consistent with the cartoon shown in Figure 9B. Analogous interpretations for particle shapes and morphologies can be made for particles with 6-, 4-, and 3-fold symmetries based on the confocal images in Figure S3 and the SEM images in Figures 8 and S11. These results also reveal that capsule/droplet structures with 3-fold symmetry are likely to have the largest capsule/droplet contact areas and the smallest droplet/water contact areas, consistent with our proposed mechanism for minimization of overall free energy.

As noted above, elastocapillarity has been leveraged in a wide range of past studies to affect transformations in soft materials systems, including studies on the elastocapillary-induced wrapping of planar polymer sheets around liquid droplets.^13,20,21^ In general, past studies on the wrapping of planar polymer sheets have involved the use of either relatively thick planar sheets or ultrathin planar sheets that are often shaped or precut to manage topological complexities associated with the wrapping of a planar sheet neatly around a droplet. The caged-droplet system investigated here differs substantially from those planar sheet systems and involves the surfactant-triggered transformation of preformed, approximately spherical, and edge-free polymer membranes encapsulating, and in contact with, microscale oil droplets. While the folding and wrapping behaviors reported here are driven by elastocapillary interactions that are, fundamentally, similar in nature to those that drive the wrapping of planar polymer sheets, this capsular system introduces geometric and topological constraints that lead to folding behaviors—and, thus, to droplet/capsule and polymerized particle shapes—that differ substantially from those reported in past studies on the wrapping of liquid droplets with planar polymer sheets.

## Summary and Conclusion

We have reported on large-scale deformations and transformations in shape that occur in polymer multilayer microcapsules partially filled with small oil droplets. These so-called “caged” oil droplets exhibit largely spherical morphologies when suspended in water (i.e., roughly spherical polymer capsules surrounding a roughly spherical oil droplet that is in contact with and partially wetting the capsule wall) but undergo folding and large transformations in shape when exposed to low concentrations of an anionic surfactant. These processes occur through a series of kinetically trapped intermediate states and, depending on the concentration of surfactant, lead to folded and polymer-wrapped oil droplets with complex anisotropic shapes and apparent six-, five-, four- and 3-fold symmetries. Folding is also reversible upon the addition of a cationic surfactant, allowing the folded cages to return to their initial spherical shapes. These processes are observed to occur only in cages that are sufficiently thin and sufficiently large in size; overall, our results support a physical picture involving surfactant-induced wetting transitions in the encapsulated oil droplets and elastocapillary deformation of the surrounding polymer shells. The use of polymerizable oils as a fugitive oil droplet component permits the anisotropic shapes of the folded and polymer-wrapped droplets to be locked in by subsequent polymerization; coupled with the use of degradable polymer cages, this approach can be used to produce solid polymer particles with complex anisotropic shapes.

Our results expand the potential utility of elastocapillarity for the synthesis of colloidal soft materials with complex shapes by using surfactants to tune droplet wetting behaviors and trigger bending transformations in thin polymer capsules, an approach that has not, to our knowledge, been used previously to control the shapes of polymer-wrapped oil droplets or design polymer particles with anisotropic shapes. In addition to the potential utility of polymer particles with complex shapes, we note that the polymer-wrapped oil droplets themselves may also be useful, particularly in the case of folded cages containing droplets of nematic LCs, for which director profiles and, thus, optical properties and other behaviors can change substantially in confined geometries. Characterization of the impacts of capsule folding and shape change on the director profiles of caged LC droplets and the synthesis of polymer particles with both anisotropic shapes and internal structures are underway and will be reported in due course.

## Supplementary Material



## Notes

Additional information includes optical, confocal fluorescence, and scanning electron microscopy images of caged LCs and polymer particles as well as plots showing changes in the distribution of shapes under different experimental conditions (PDF)

## ∥

Dept. of Chemical and Biomolecular Engineering, Cornell Univ., Ithaca, NY 14853, USA
