# Supplementary material for: Polymer-Wrapped Oil Droplets and Polymer Particles with Complex Curvilinear Polyhedral Geometries by Surfactant-Driven Elastocapillary Buckling of Polymer Capsules
Source: Chem Mater. 2025 Oct 29;37(21):8664–76. doi: 10.1021/acs.chemmater.5c01556 (PMC12613320; doi:10.1021/acs.chemmater.5c01556)
Supplement: Supplementary file 1 [file cm5c01556_si_001.pdf]

Polymer-Wrapped Oil Droplets and Polymer Particles with Complex Curvilinear Polyhedral Geometries by Surfactant-Driven Elastocapillary Buckling of Polymer Capsules

Xuanrong Guo,[1] Saverio E. Spagnolie,[3] Nicholas L. Abbott, [1,‡,*] and David M. Lynn[1,2,*]

[1]*Department of Chemical and Biological Engineering, 1415 Engineering Drive, University of Wisconsin – Madison, Madison, Wisconsin 53706, USA,* [2]*Department of Chemistry, 1101 University Avenue, University of Wisconsin – Madison, Madison, Wisconsin 53706, USA, and* [3]*Department of Mathematics, 505 Van Vleck, University of Wisconsin – Madison, Madison, Wisconsin 53706, USA.* ‡*Current address: Dept. of Chemical and Biomolecular Engineering, Cornell Univ., Ithaca, NY 14853, USA; E-mail: nla34@cornell.edu (N.L.A.); david.lynn@wisc.edu (D.M.L.)*

SUPPORTING INFORMATION

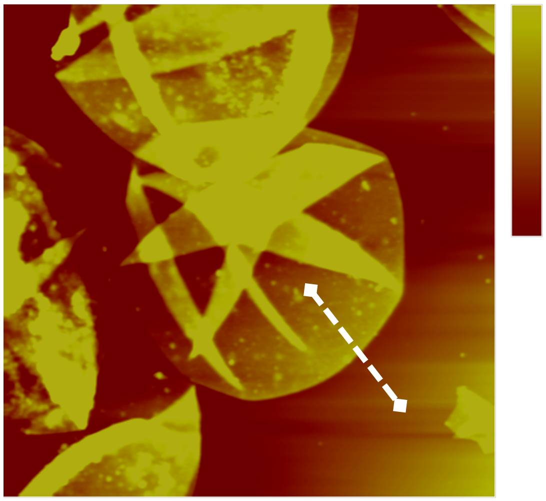

Figure S1: 50 μm x 50 μm tapping mode atomic force microscopy (AFM) image of dried PEI/PVDMA capsules four bilayers thick. The image was acquired using a Multimode IIIa instrument in contact mode with a 1 Hz scan rate. Hollow capsules suspended in water, prepared as described in the main text, were deposited on a silicon substrate and dried under vacuum overnight. Vertical height profiles across the capsule rim and adjacent substrate baseline, in the approximate location marked by the white dotted line, were extracted and divided by two to determine the wall thicknesses, as described previously in: Hirotaka Ejima *et al.* One-Step Assembly of Coordination Complexes for Versatile Film and Particle Engineering. *Science,* 341,154-157 (2013). Wall thicknesses were averaged across measurements for N = 50 capsules to yield an average capsule wall thickness of 54.9 ± 7.4 nm. The color scale at the upper right (brown to yellow) ranges from 0 to 200 nm.

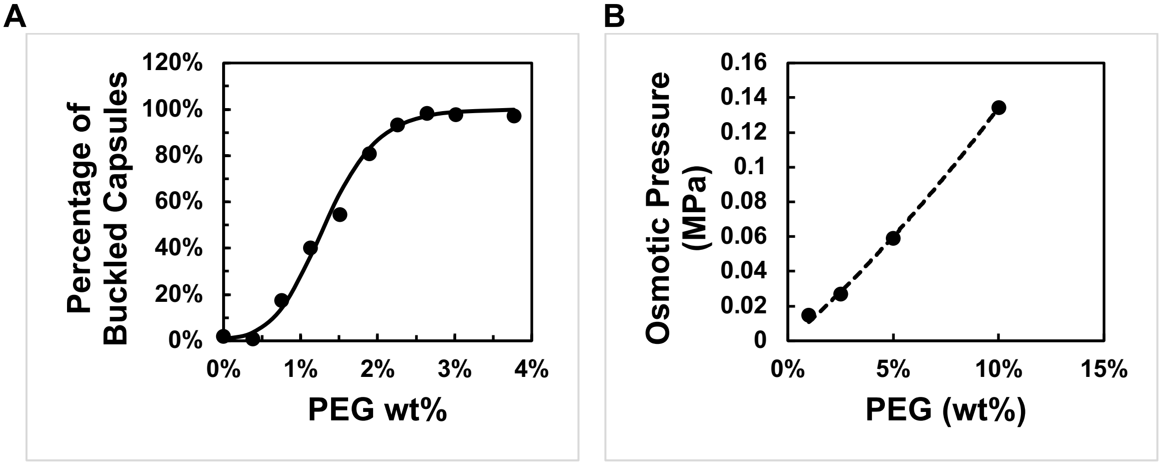

Figure S2: Measurement of shear modulus by the osmotic-induced buckling method. A) Percentage of deformed capsules (N > 300 capsules for each data point) as a function of PEG concentration. A sigmoid function was used to fit the curve and determine the critical PEG concentration. B) Standard osmotic pressure calibration curve used to estimate *Pc (*~ 0.015 MPa). The average shear modulus of the capsules (μ) was determined to be 38 MPa. See information shown below for additional experimental details.

Osmolalities were measured using a Wescor Vapro 5520 vapor-pressure osmometer (Wescor, Logan, UT). The osmotic pressure of polyethylene glycol (PEG, MW ~20,000, PDI ~1.13) solutions at different concentrations was calculated using Equation S1:

$$\text{Osmotic Pressure (MPa)} = \text{Osmolality} \times \text{density} \times \text{RT} = \frac{\text{Osmolality}}{\text{mol/kg}} \times 2.46\ \text{MPa} \qquad \text{(S1)}$$

The elasticity of hollow PEI/PVDMA capsules was assessed by the osmotic-induced buckling method, as previously reported in: Gao, C., Donath, E., Moya, S. *et al.* Elasticity of hollow polyelectrolyte capsules prepared by the layer-by-layer technique. *Eur. Phys. J. E,* **5**, 21–27 (2001). Based on the assumption that high MW polymers would be unable to diffuse through the capsule wall, suspensions of spherical hollow capsules were exposed to a series of PEG solutions to create an osmotic pressure gradient. The percentage of deformed capsules was calculated and plotted against PEG concentration (as shown in panel A), and the critical concentration corresponding to 50% capsule invagination was determined. A standard calibration curve was then generated to convert this critical PEG concentration to critical osmotic pressure, P_C. Assuming that the stretching of the crosslinked capsule wall was negligible under these conditions, P_C is related to the shear modulus of capsule wall (μ), wall thickness (δ), and capsule radius (R) according to Equation S2:

$$P_C = 4\mu\left(\frac{\delta}{R}\right)^2 \qquad \text{(S2)}$$

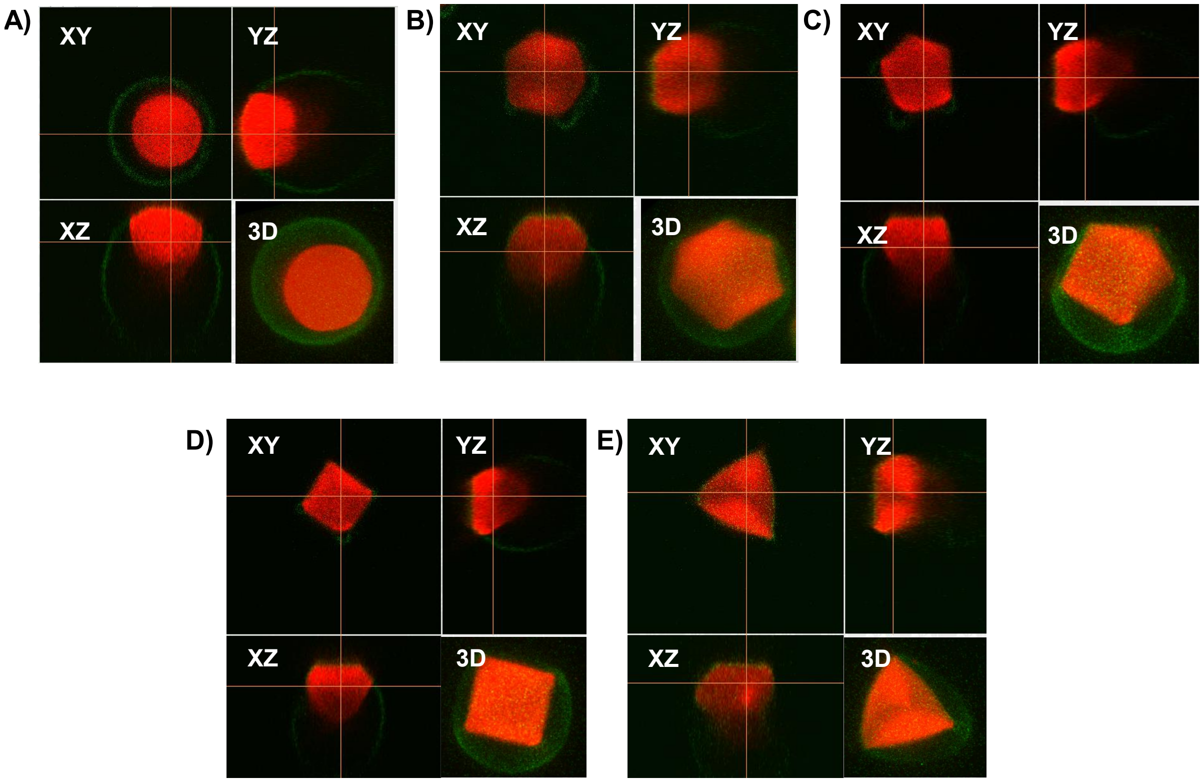

Figure S3: Representative confocal microscopy images of 'caged' LC droplets suspended in 1 μM SDS. A spherical 'caged' droplet (A) and droplets with 6-fold (B), 5-fold (C), 4-fold (D), and 3-fold (E) symmetries are shown.

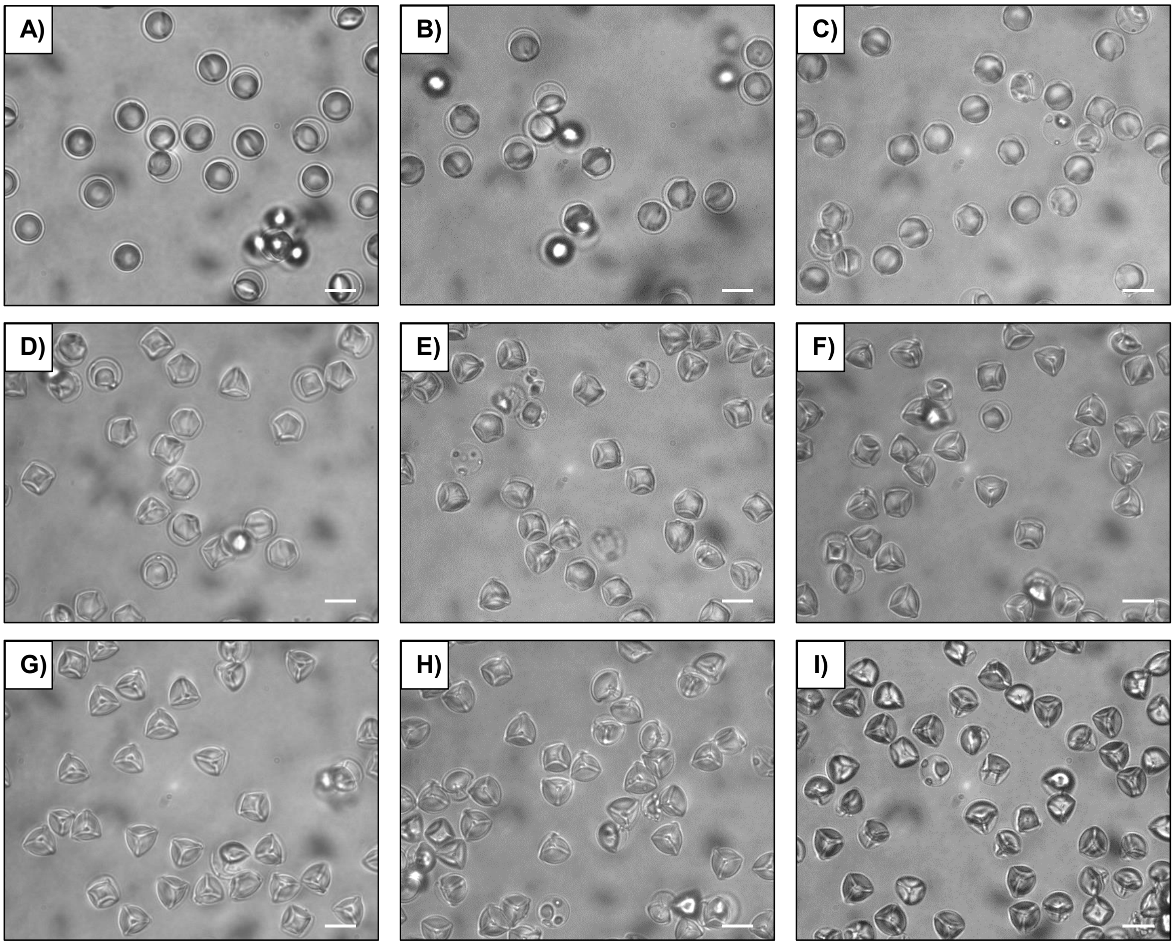

Figure S4: Bright-field microscopy images showing distributions of ‘caged’ LC droplets with different apparent shapes in (A) 0 μM SDS, (B) 100 nM SDS, (C) 500 nM SDS, (D) 1 μM SDS, (E) 2.5 μM SDS, (F) 5 μM SDS, (G) 10 μM SDS, (H) 20 μM SDS, and (I) 1 mM SDS. Scale bars are 10 μm.

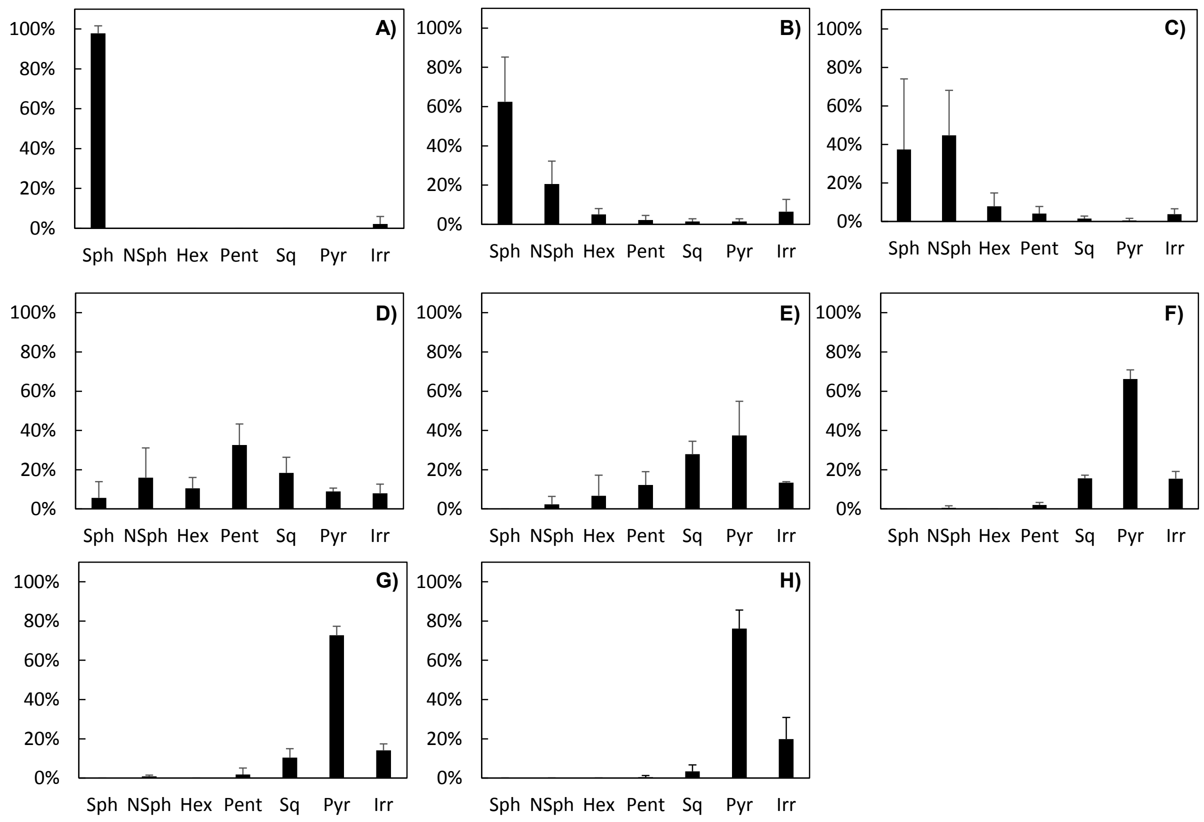

Figure S5: Plots showing distributions of ‘caged’ LC droplets with different apparent shapes in (A) 0 μM SDS, (B) 100 nM SDS, (C) 500 nM SDS, (D) 1 μM SDS, (E) 2.5 μM SDS, (F) 5 μM SDS, (G) 10 μM SDS, and (H) 20 μM SDS; Sph = spherical; NSph = non-spherical; Hex = hexagon; Pent = pentagon; Sq = square; Pyr = pyramid; Irr = irregular shaped. Panels A, D, and G shown here correspond to panels A, C, and E of Figure 3 also shown in the main text. The number of droplets measured was N > 150 for each plot. Error bars are standard deviations of three parallel samples.

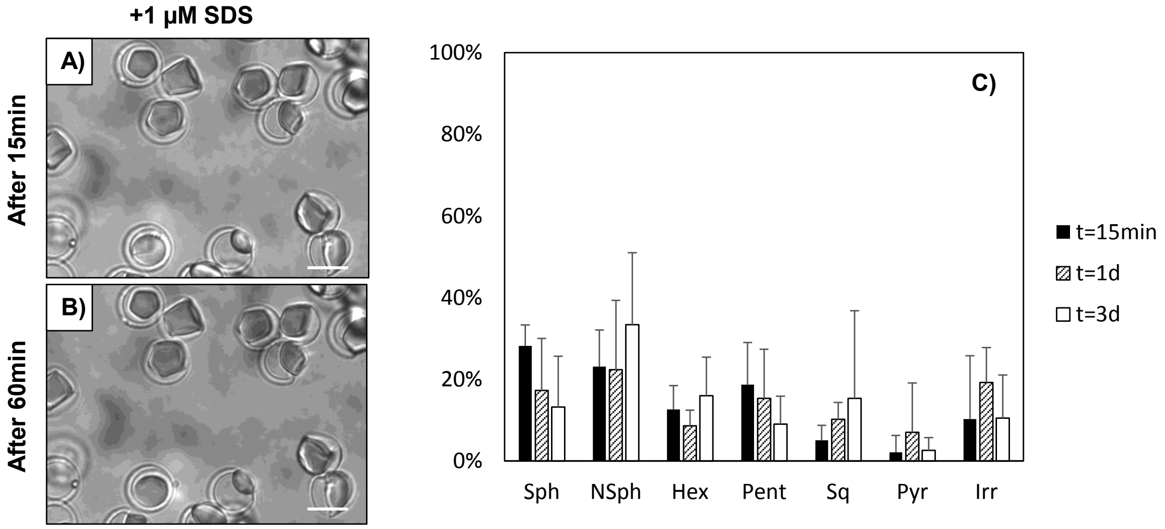

Figure S6: (A-B) Bright-field images showing surface immobilized ‘caged’ LC droplets after exposure to 1 µM SDS for (A) 15 min and (B) 45 min. (C) Plot showing distributions of ‘caged’ LC droplets with different apparent shapes suspended in 1 µM SDS after incubation for 15 min (black), 1 day (diagonal hashes) and 3 days (white); Sph = spherical; NSph = non-spherical; Hex = hexagon; Pent = pentagon; Sq = square; Pyr = pyramid; Irr = irregular shaped. The number of droplets measured was N > 100 for each time point. Error bars are standard deviations of three parallel samples. Scale bars are 10 µm.

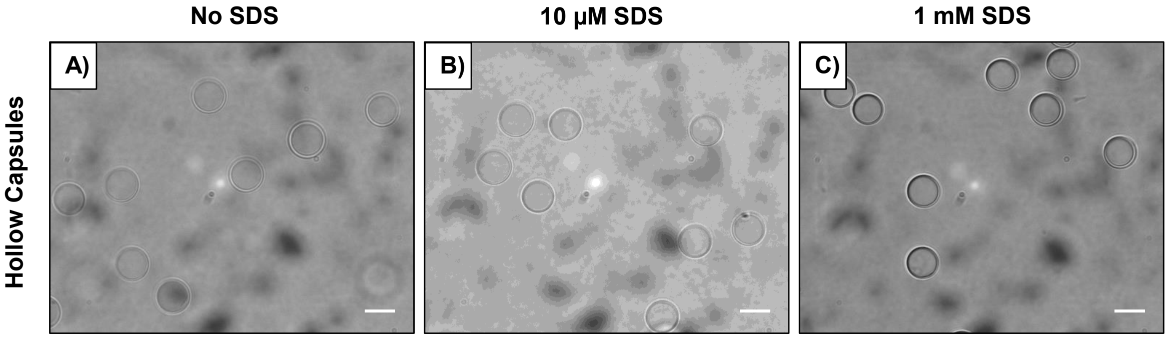

Figure S7: Bright-field images showing empty 4.5 bilayer '8-µm-large' capsules (no infused oils) after exposure to (A) 0 µM SDS, (B) 10 µM SDS, and (C) 1 mM SDS for 15 min. Scale bars are 10 µm.

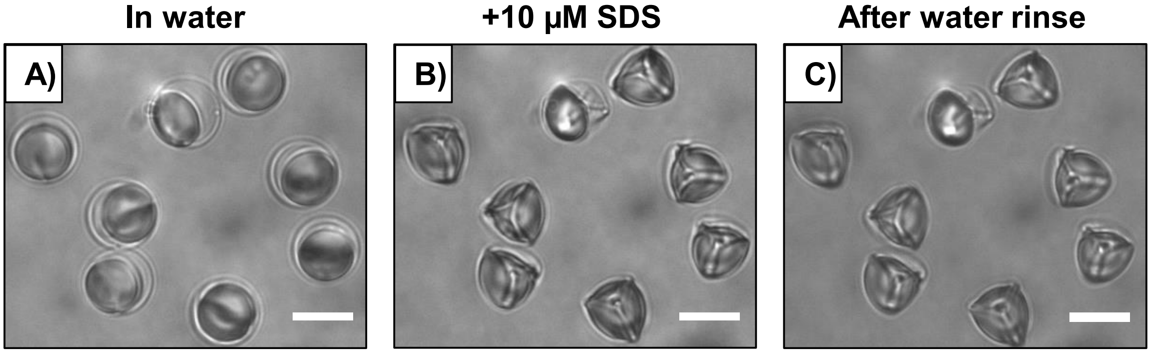

Figure S8: Bright-field images showing surface immobilized 'caged' LC droplets (A) in 0 µM SDS, (B) after exposure to 10 µM SDS, and (C) after rinsing and replacing with water for 30 min. Scale bars are 10 µm.

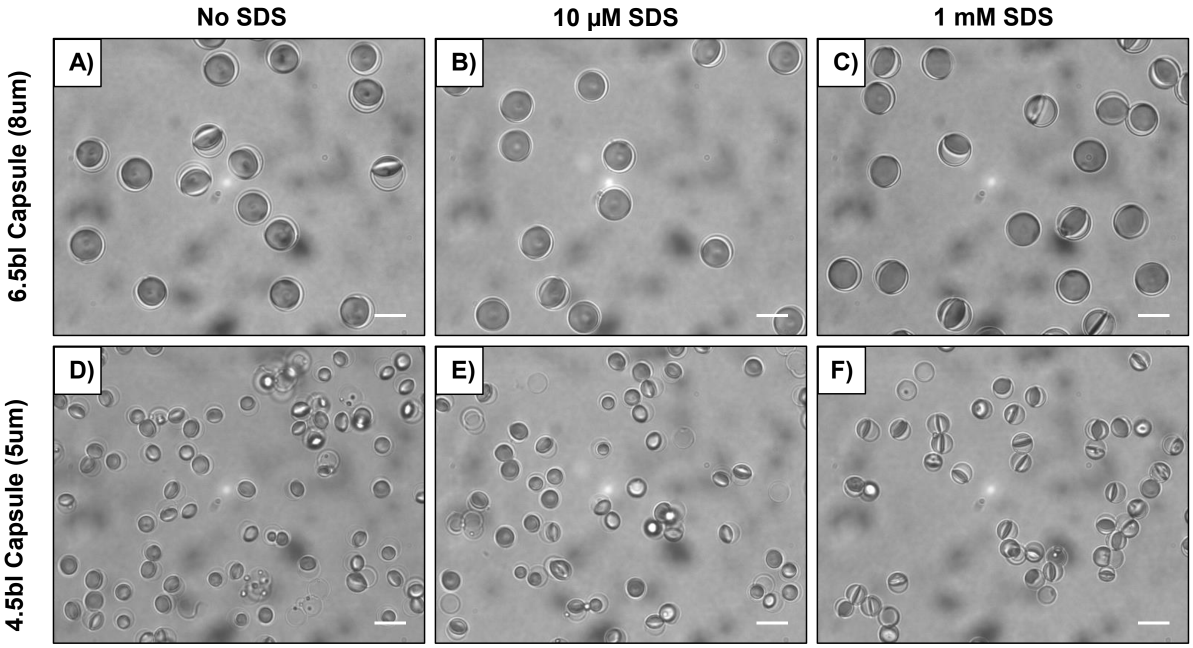

Figure S9: Bright-field images showing LC droplets 'caged' in 6.5 bilayer '8-µm-large' capsules (A-C) and 4.5 bilayer '5-µm-small' capsules (D-F) after exposure to (A, D) 0 µM SDS, (B, E) 10 µM SDS, and (C, F) 1 mM SDS for 15 min. Scale bars are 10 µm.

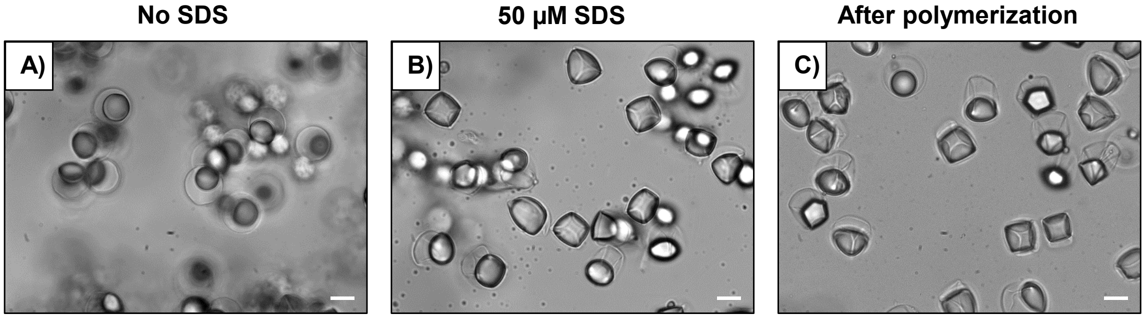

Figure S10: (A) Representative bright-field microscopy images of polymerizable oils 'caged' in degradable polymer capsules. (B-C) Polymerizable oils 'caged' in degradable polymer capsules shown in the presence of 50 µM SDS before (B) and after (C) polymerization. Scale bars are 10 µm.

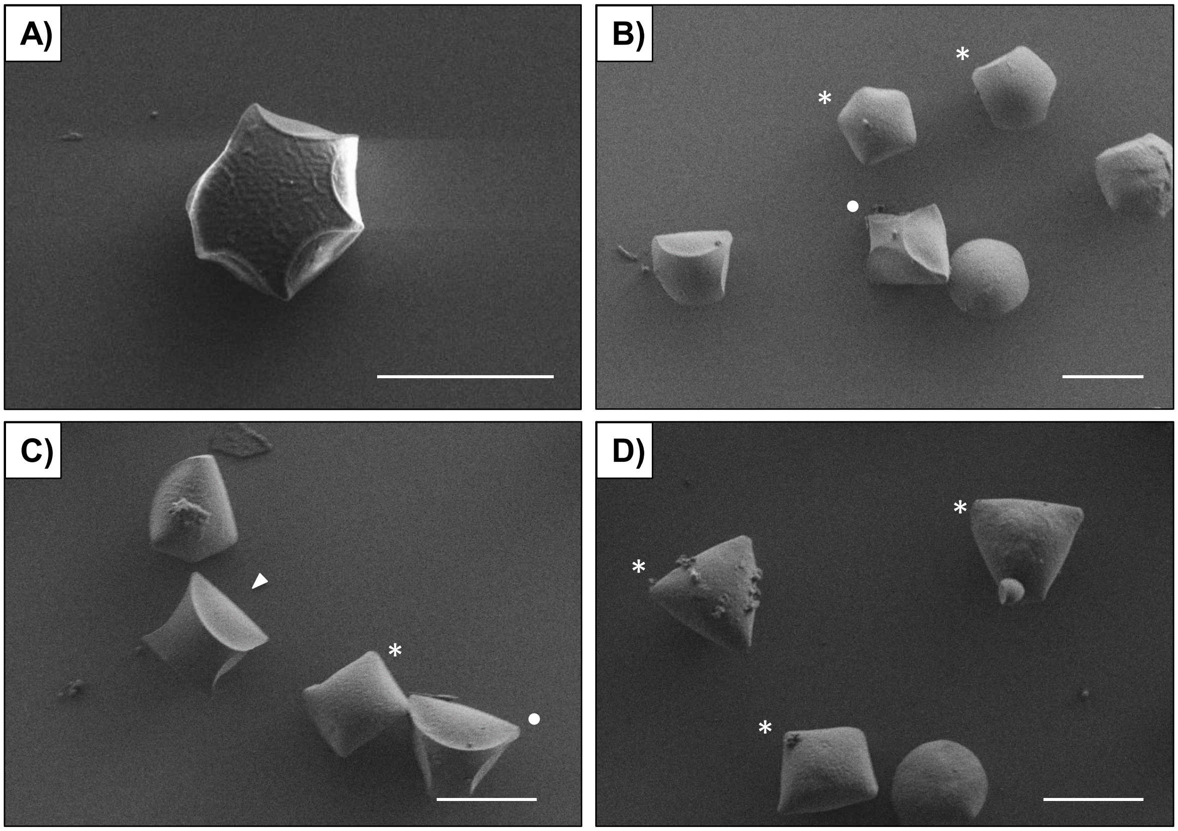

Figure S11: Additional SEM images showing morphologies of 'cage-free' polymerized particles after treatment with 10 mM DTT. A polymer particle with apparent 6-fold symmetry is shown in (A). Particles appeared to be trapped in intermediate states are labeled with white dots. Particles showing 'top surfaces' (arrowhead) and 'bottom surfaces' (asterisks), as defined in the main text, are also shown. Scale bars are 10 μm.
